# Rodent Models for the Study of Soil-Transmitted Helminths: A Proteomics Approach

**DOI:** 10.3389/fcimb.2021.639573

**Published:** 2021-04-22

**Authors:** Karen J. Montaño, Carmen Cuéllar, Javier Sotillo

**Affiliations:** ^1^ Centro Nacional de Microbiología, Instituto de Salud Carlos III, Madrid, Spain; ^2^ Departamento de Microbiología y Parasitología, Facultad de Farmacia, Universidad Complutense, Madrid, Spain

**Keywords:** proteomics, soil-transmitted helminths (STHs), host-parasite interactions, *Nippostrongylus brasiliensis*, *Heligmosomoides polygyrus*, *Trichuris muris*, vaccines, immunomodulation

## Abstract

Soil-transmitted helminths (STH) affect hundreds of millions worldwide and are some of the most important neglected tropical diseases in terms of morbidity. Due to the difficulty in studying STH human infections, rodent models have become increasingly used, mainly because of their similarities in life cycle. *Ascaris suum* and *Trichuris muris* have been proven appropriate and low maintenance models for the study of ascariasis and trichuriasis. In the case of hookworms, despite most of the murine models do not fully reproduce the life cycle of *Necator americanus*, their proteomic similarity makes them highly suitable for the development of novel vaccine candidates and for the study of hookworm biological features. Furthermore, these models have been helpful in elucidating some basic aspects of our immune system, and are currently being used by numerous researchers to develop novel molecules with immunomodulatory proteins. Herein we review the similarities in the proteomic composition between *Nippostrongylus brasiliensis*, *Heligmosomoides polygyrus bakeri* and *Trichuris muris* and their respective human counterpart with a focus on the vaccine candidates and immunomodulatory proteins being currently studied.

## Introduction

Infection by soil-transmitted helminths (STHs), some of the most common neglected tropical parasites in the world, affects mainly low and middle-income countries (Brooker, 2010). Indeed, it is considered that, globally, nearly 2 billion people are infected with STHs (Brooker, 2010; World Health Organization, 2012), and hookworm infection alone results in >4 million disability-adjusted life years lost annually (DALYs), as well as in significant economic losses (Bartsch et al., 2016). *Ascaris lumbricoides*, *Trichuris trichiura*, and hookworm (mainly *Necator americanus* and *Ancylostoma duodenale*) are the most common species that infect humans (Jourdan et al., 2018). Although competent health care and wide use of available anthelmintic drugs are currently the main approaches for the elimination of most helminth infections, their efficacy varies and chemotherapy does not prevent reinfection (Loukas et al., 2016); thus, it becomes necessary to continue our efforts to improve our understanding of these parasitic diseases. Due to the limited availability and difficulty in obtaining parasite material, researchers have widely used different animal models that share similarities in the life cycle, immune response elicited or both with their human counterpart (Scott and Tanguay, 1994; Camberis et al., 2003). In this regard, rodent models are, by far, the most popular and frequently used animal models and have been helpful in characterizing many aspects of human helminth infection.

*N. americanus*, one of the most important STHs in terms of morbidity, can survive for decades in the small intestine of their human hosts (Loukas et al., 2016). While *N. americanus* is notably common in most of Africa, southern China, Southeast Asia and the Americas, *A. duodenale* is endemic in northern regions of India and China, in the Mediterranean region and in North Africa. Furthermore, in some parts of Africa, China and India, it is not unusual to observe mixed human infections with *N. americanus* and *A. duodenale* (Pullan et al., 2014). The life cycle of this group of nematodes is very complex, and involves free-living and parasitic stages as well as an intraorganic migration in the definitive host. Hookworm eggs hatch in soil and released rhabditiform larvae moult twice before becoming filariform and infective (iL3). iL3s penetrate the skin of the host and are carried through the bloodstream first to the heart and then to lungs. Following exit from the alveolar capillaries, iL3s ascend the bronchial tree to reach the pharynx and are swallowed. Finally, hookworms complete their migration to the small bowel, typically the distal jejunum, where immature L5 hookworms attach themselves in position to feed and avoid ejection by gut peristalsis (Loukas et al., 2016).

Interestingly, a hamster model susceptible to *N. americanus* is available; however, although adult worms can fully develop without the requirement of corticosteroids, this model was developed after decades of passaging through immunosuppressed hamsters (Jian et al., 2003; Xiao et al., 2008), and the extent of adaptation and genomic and proteomic differences with worms obtained from the human host is yet to be determined. Indeed, worms obtained from hamsters are smaller in size, less fertile and infections do not last longer than a few months (Jian et al., 2003; Xiao et al., 2008), although they do elicit a protective immunity similar to that observed in the related canine hookworm species *Ancylostoma caninum*. This model has also proved useful for the screening of vaccine candidates and the assessment of antihelminthic drugs (Xue et al., 2005; Xiao et al., 2008; Xue et al., 2010; Zhan et al., 2010), however, the impossibility to use hamsters in some countries (e.g. Australia) and the low availability of molecular biology reagents for hamsters can make it challenging to work with.

Because of this, different animal models have been used to study hookworm-host interactions, including the related ancylostomatids *A. caninum* in dogs (Shepherd et al., 2018) and *A. ceylanicum* in hamsters (Alkazmi and Behnke, 2010; Traub, 2013), as well as the murine nematodes *Nippostrongylus brasiliensis* and *Heligmosomoides polygyrus bakeri* (both belonging to the Trichostrongyloidea superfamily), all part of the clade V of nematodes. This clade contains members of the suborder Rhabditina with nematodes from the Strongylida and other orders (Blaxter et al., 1998). *A. ceylanicum* infection is a zoonotic disease, and can produce symptomatic infections in humans (Loukas et al., 2016). It can also infect hamsters, where it develops patent infections (Alkazmi and Behnke, 2010), and can elicit acquired immunity, making it a suitable model for the study of hookworm infections (Loukas et al., 2016) with the same limitations listed above.

*N. brasiliensis*, a rodent strongyle nematode widely used by parasitologists, has a similar life cycle to *N. americanus*, including skin penetration, migration through the lungs and establishment in the small intestine of its host, although it is rapidly eliminated and does not recapitulate the long-lasting infections found with *N. americanus* (Camberis et al., 2003). Furthermore, *N. brasiliensis* induces a Th2 type immune response that manifests all the characteristics of a human hookworm infection, including IgE production and eosinophilia, which drive pathology in some allergic diseases (Nair and Herbert, 2016), as well as mastocytosis and mucus production (Camberis et al., 2003). Researchers have taken advantage of the similar life cycle and immunological responses between *N. brasiliensis* and *N. americanus* to conduct immunological studies (both systemic and mucosal) aimed at studying the mechanisms involved in human hookworm infections (Nair and Herbert, 2016).

Despite *H. polygyrus bakeri* does not infect through the skin or migrate through the lungs (as it depends on oral ingestion of infective larvae), it has been extensively employed as a model for human hookworm infections. Indeed, similarly to hookworms, *H. polygyrus bakeri* induces chronic intestinal infections in several mice strains, and the modified Th2 cell responses induced by infection (a Th2-like response linked with the production of anti-inflammatory cytokines and Treg activity) does not completely eliminate the parasites (Wells and Behnke, 1988; Maizels, 2005; Bungiro et al., 2008; Reynolds et al., 2012; Nair and Herbert, 2016). Furthermore, the study of *H. polygyrus bakeri* and *N. brasiliensis* infection in rodents has provided the immunology community with important information about the humoral and cellular mechanisms involved in the induction and development of Th2 immune responses and their capacity in protecting against helminth infections (Ogilvie and Jones, 1971; Ishizaka et al., 1976; Urban et al., 1991; Camberis et al., 2003).

In addition to hookworms, infection with whipworms (mainly *T. trichiura*) largely contributes to the pathological burden caused by STHs. More than 70 species of *Trichuris* (including worms of veterinary, scientific and human interest) have been described so far (Hurst and Else, 2013). All these species were classified within clade I, which groups vertebrate-parasites from the order Trichocephalida together with insect and plant-parasitic nematodes (Blaxter et al., 1998). Due to the difficulty in obtaining live worms from infected people and the impossibility of maintaining *T. trichiura* in the laboratory, *Trichuris muris* has become a widely used laboratory model being physiologically, morphologically, and antigenically similar to the human whipworm species (Grencis, 1993; Dixon et al., 2008). Indeed, the *T. muris* model has allowed researchers to understand relevant features concerning immunity to gut-dwelling nematode parasites as well as to gain a better knowledge of the immune system (Hurst and Else, 2013). Furthermore, the knowledge attained from this animal model has been applied to better understand human Trichuriasis (Faulkner et al., 2002) and other intestinal helminth infections (Turner et al., 2003).

Ascariasis, mainly caused by *A. lumbricoides*, affects over 800 million people worldwide (Pullan et al., 2014). Similarly to what occurs with other STHs, it is highly challenging to obtain adult worms, and model organisms have been developed (Holland, 2013). The related species *A. suum* is a natural parasite of pigs; however this animal model has not been widely used because of its cost, large size and difficult husbandry (Holland, 2013). This species was found to be able to infect mice and to follow a similar infection behaviour as the one observed in its natural hosts (Slotved et al., 1998), and further research identified mouse strains with different compatibility (e.g. the susceptible C57BL/6 and the resistant CBA/Ca strains), providing a convenient model to investigate the basis of Ascaris biology and for the development of vaccine candidates (Lewis et al., 2007; Deslyper et al., 2019).

Despite the significant advantages of murine models in terms of reproductive capacity, handling, and costs, there are other models used for the study of helminth infections such as the pig whipworm *Trichuris suis*, which pathophysiology is very similar to that occurring in human infections (Dawson et al., 2020) or, as mentioned above, the dog hookworm *A. caninum* (Shepherd et al., 2018). Nevertheless, due to ethical considerations, complex logistics and cost, pig and dog models are less used in parasitological research and will not be the scope of this review.

The need to develop novel and effective treatments against STH is indisputable, and rodent models can provide important information. Understanding, not only the immunological, physiological, anatomical and metabolic similarities that each model has, but also the proteomic and genomic similarities between all species is key for the design of appropriate control approaches. In this review, we compare the available proteomic data between STH of human importance and their murine model counterparts with a focus on the characterization of vaccine candidates and immunomodulatory molecules. This analysis provides the first step towards a rational selection of the most appropriate model for the analysis of a particular protein candidate; however, ideally, a combined approach integrating different transcriptomic, proteomic, lipidomic and metabolomic information will provide a more comprehensive picture of the suitability of a particular model.

## Genomic and Proteomic Information From Animal Models

During the last decades, one of the major caveats in the study of host-hookworms interactions has been the lack of comprehensive and thoroughly annotated genomic and proteomic databases. However, in the recent years, the development of novel sequencing platforms and more sensitive mass spectrometers, as well as different initiatives (i.e., 50 helminth genomes project; https://www.sanger.ac.uk/science/collaboration/50hgp) have provided useful information (Sotillo et al., 2017). In the case of *N. americanus*, the first draft genome was published in 2014 (Tang et al., 2014), and a more comprehensive genome version annotated using proteomic and transcriptomic data has recently been published (Logan et al., 2020). Similarly, the genomes and transcriptomes for the whipworms *T. trichiura* and *T. muris* were published in 2014 (Foth et al., 2014). In the case of *A. lumbricoide*s, the Parasite Genomics group at the Wellcome Trust Sanger Institute performed genome predictions as part of the 50 helminth genomes project (International Helminth Genomes Consortium, 2019).

**Figure 1:**
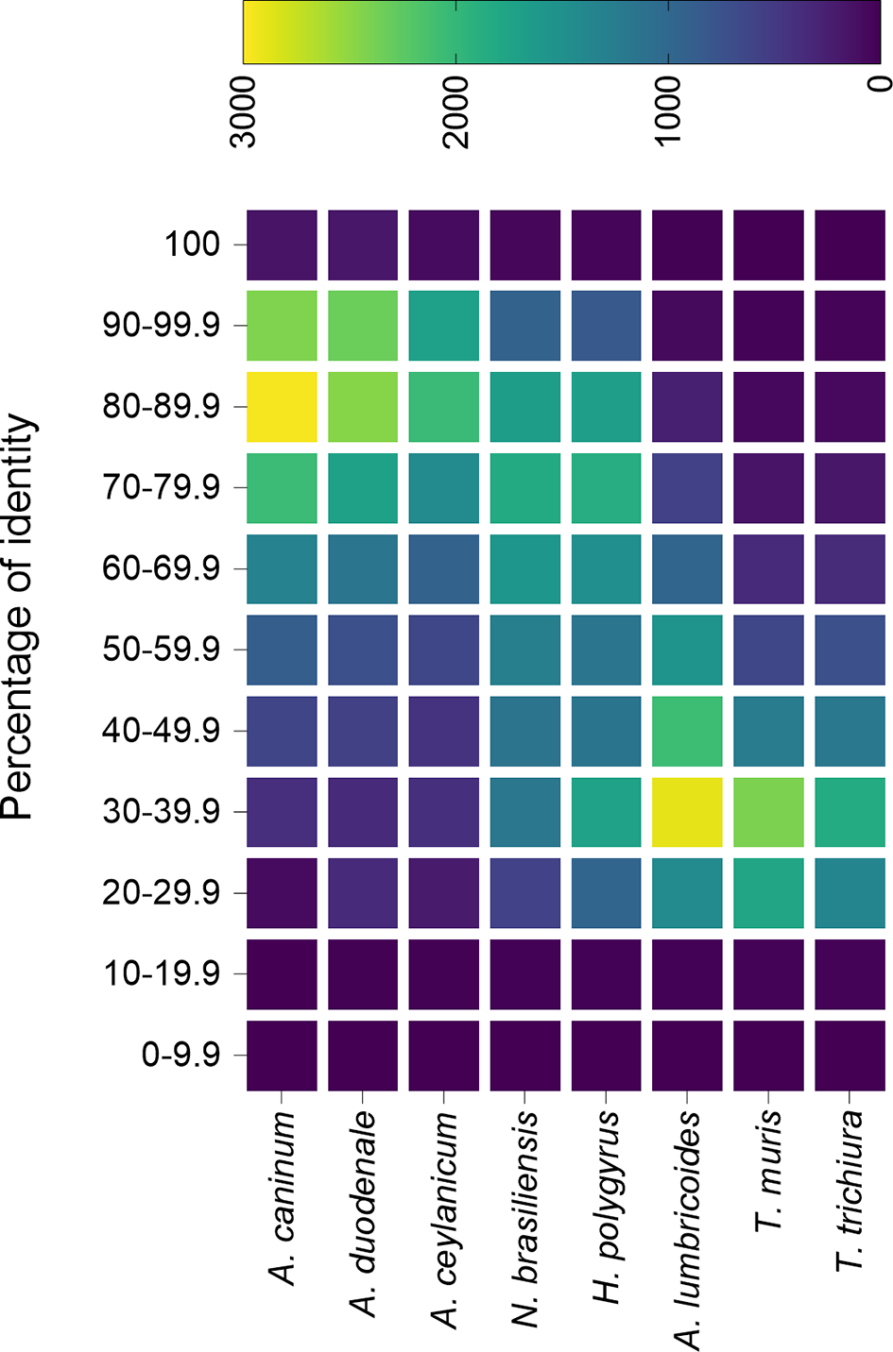
Percentage identity of *Necator americanus* predicted proteome with the predicted proteins from different nematode species. The predicted proteome from different species of hookworm, hookworm mouse models and unrelated nematodes were compared against the predicted proteome from *N. americanus* and plotted in a heatmap. All predicted proteomes were downloaded from Parasite WormBase (v.14.0) and protein identity was calculated using Blast. Colour represents the number of proteins within a range of identity percentage.

A similarity analysis between the predicted proteome from *N. americanus* and other nematodes from the Ancylostomatidae family as well as hookworm models and other nematodes (all data downloaded from ParasiteWormBase v.14.0) shows that *A. caninum*, *A. duodenale* and *A. ceylanicum* proteins are, in general, more similar to *N. americanus* proteome (**Figure 1**). This analysis also showed that despite *N. brasiliensis* and *H. polygyrus bakeri* do not belong to the Ancylostomatidae family and are, thus, less related to hookworm, they share a high degree of similarity (>65%) in their proteome with *N. americanus* (**Figure 1**), compared to other nematodes. Despite the limitations of analysing the proteins only at the amino acid level, it is well accepted that proteins sharing over 40% (60% in the case of enzymes) sequence identity might share similar functions (Rost, 1999; Tian and Skolnick, 2003).

This is in agreement to what has been found recently, where *N. americanus* and hookworm animal models contained a similar number of predicted proteins encoded by their genomes, and proteins actively secreted by their adult stages presented a similar protein family profile (Logan et al., 2020). Indeed, from the 198 proteins secreted by *N. americanus* adult worms, 173 (>87%) contained homologs in the secretomes from *H. polygyrus bakeri*, *N. brasiliensis* and *A. caninum* (Logan et al., 2020). One of the most represented families in the secretomes of these adult worm species is the sperm-coating protein (SCP)-like extracellular proteins, also called SCP/Tpx-1/Ag5/PR-1/Sc7 (SCP/TAPS; Pfam accession number no. PF00188). A total of 51 out of the 54 SCP/TAPS proteins found in the secretome of *N. americanus* had homologs in *H. polygyrus bakeri*, *N. brasiliensis* and *A. caninum*, which highlights the usefulness of using these murine models to study this particular family of proteins. Despite a phylogenetic analysis showed *N. americanus* SCP/TAPS proteins cluster more with *A. caninum* proteins than with *N. brasiliensis* or *H. polygyrus bakeri*, there are strong clade-specific similarities (Logan et al., 2020), and the high degree of diversity in the evolution of SCP/TAPS was speculated to be related to host-specific roles for this family of proteins (Logan et al., 2020).

Proteases (aspartyl-, cystein-, metallo- and serine-proteases) are also highly abundant in the secretome of *N. americanus* adult worms (Logan et al., 2020), as well as the murine models (Hewitson et al., 2011; Sotillo et al., 2014). A homology analysis showed that proteases secreted by *N. americanus* had, in general, a higher degree of homology to those from the *H. polygyrus bakeri* and *N. brasiliensis* rather than *A. caninum* (Logan et al., 2020), which would make these models highly suitable for the development of vaccine studies as discussed in the next section.

**Figure 2:**
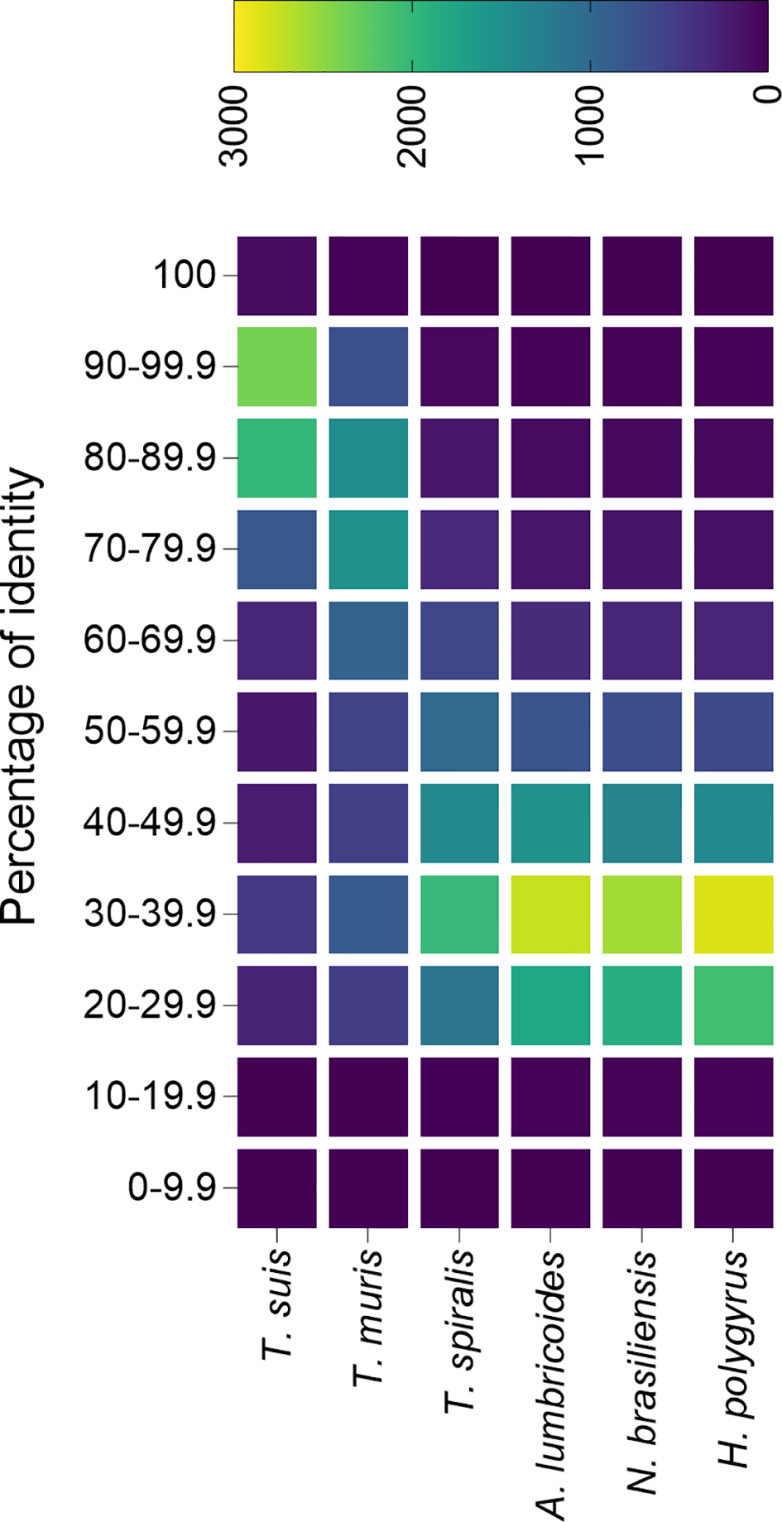
Percentage identity of *Trichuris trichiura* predicted proteome with the predicted proteins from different nematode species. The predicted proteome from different species of trichurids and unrelated nematodes were compared against the predicted proteome from *T. trichiura* and plotted in a heatmap. All predicted proteomes were downloaded from Parasite WormBase (v.14.0) and protein identity was calculated using Blast. Colour represents the number of proteins within a range of identity percentage.

Foth et al. sequenced and assembled the genome from both *T. trichiura* and *T. muris*, and found that most Trichuris genes are orthologs shared by both species (Foth et al., 2014). Furthermore, predicted proteomes are highly similar, with over 5,000 proteins having an average homology of 79% and only 2,350 and 3,817 proteins specific from *T. trichiura* and *T. muris* respectively, which highlights the usefulness of the mouse model to study human whipworm infections (Foth et al., 2014). An analysis of the similarity between the *T. trichiura* predicted proteome and other trichurids (i.e. *T. suis* and *T. muris*) as well as unrelated nematodes (all data downloaded from ParasiteWormBase v.14.0) confirms that proteins from both the pig and mouse models are highly similar to the human whipworm, and could be useful for the study of whipworm infections (**Figure 2**).

Only two studies have attempted to characterize the proteins secreted by *T. muris*, identifying 148 (Eichenberger et al., 2018b) and 73 (Tritten et al., 2017) proteins, while in the case of *T. suis* 328 proteins were identified (Leroux et al., 2018). The lower number of identified proteins in the mouse model in comparison with *T. suis* could be a reflection of the more stringent database search settings used. For instance, while Tritten et al. and Eichenberger et al. included databases from the parasite and the host (to eliminate host-associated proteins) and only proteins identified with two or more peptides were used for further analysis, Leroux et al. only used a parasite database (no contaminants were included in the search) and proteins identified with only one peptide were considered as valid identifications (Tritten et al., 2017; Eichenberger et al., 2018b; Leroux et al., 2018). It is noteworthy the low number of SCP/TAPS proteins identified in the *T. muris* secretome compared to parasites from clade V, which agrees with previous observations where this family of proteins is significantly expanded in clades IVa and V but not in clade I (Wilbers et al., 2018; International Helminth Genomes Consortium, 2019). To elucidate the degree of similarity between animal models and human whipworm infections, a comparative analysis of the secretomes from all three parasites would be of high interest, although the difficulty in obtaining viable worms from the human host makes this type of analysis currently very challenging.

## Development of Vaccine Candidates in Murine Models

Since resistance to different antihelminthic drugs is being widely reported in human and animal nematodes, there is an urgent need for vaccines that could complement the current approach to helminth control. In this regard, the different rodent models used to study STHs could be of importance. Indeed, both hookworm hamster models (*N. americanus* and *A. ceylanicum*) have been used for the screening of vaccine candidates (Ghosh et al., 2006; Bungiro et al., 2008; Zhan et al., 2010), and *A. ceylanicum* has been proven a good model for selection of vaccine candidates using bioinformatic and functional approaches (Wei et al., 2016), providing important information for the development of these candidates.

**Figure 3:**
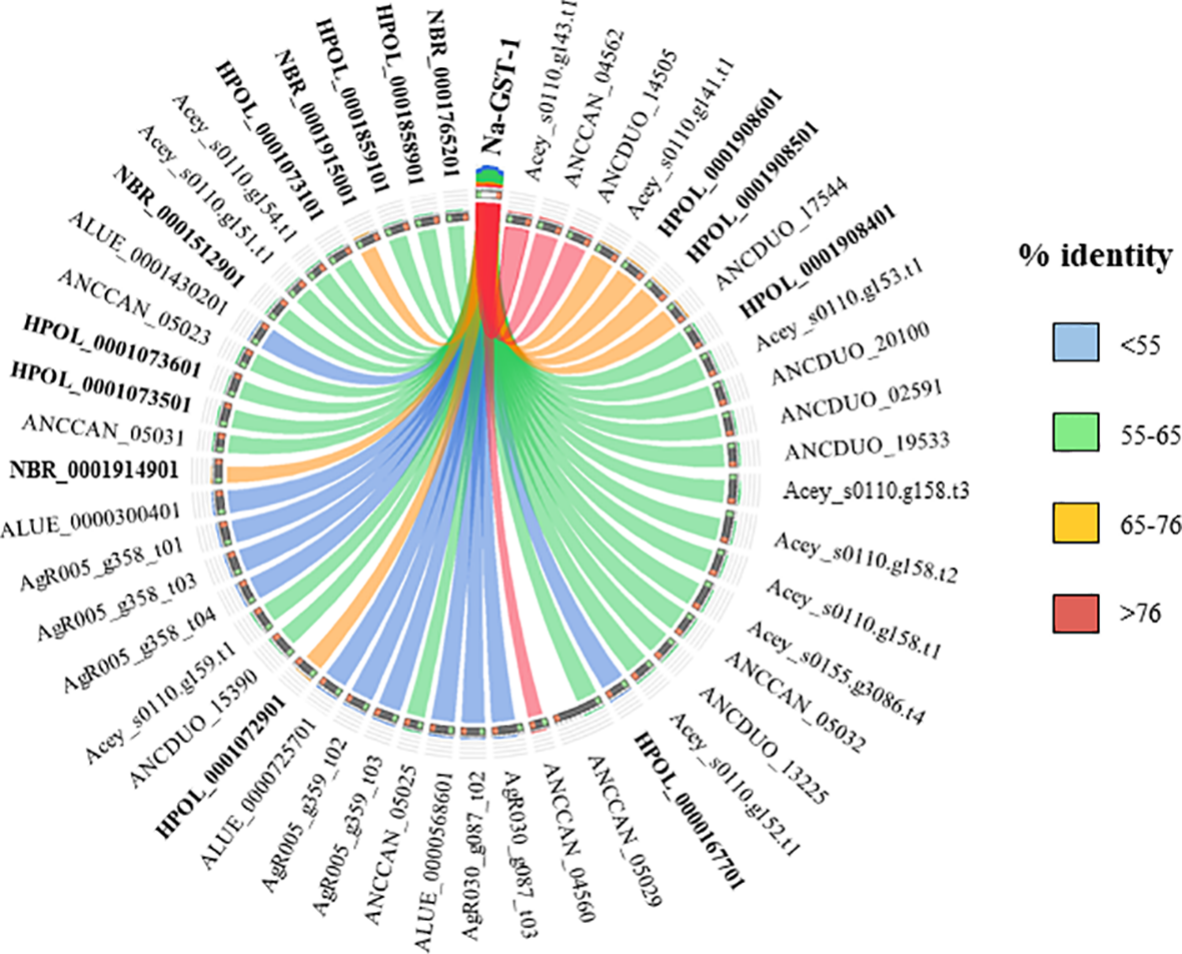
Similarity plot. Circos plot generated using Circoletto (Darzentas, 2010) showing the percentage of identity between Na-GST-1 and their homologues in different rodent model nematodes. Only homologues with e-values < 1E-50 are shown. Protein names as per Parasite WormBase database (v.15) have been used for comparison. ALUE, *Ascaris lumbricoides*; AgR, *Ascaris suum*; ANCCAN, *Ancylostoma caninum*; Acey, *Ancylostoma ceylanicum*; ANCDUO, *Ancylostoma duodenale*; HPOL, *Heligmosomoides polygyrus*; NBR, *Nippostrongylus brasiliensis*; TMUE, *Trichuris muris*; TTRE, *Trichuris trichiura*.

**Figure 4:**
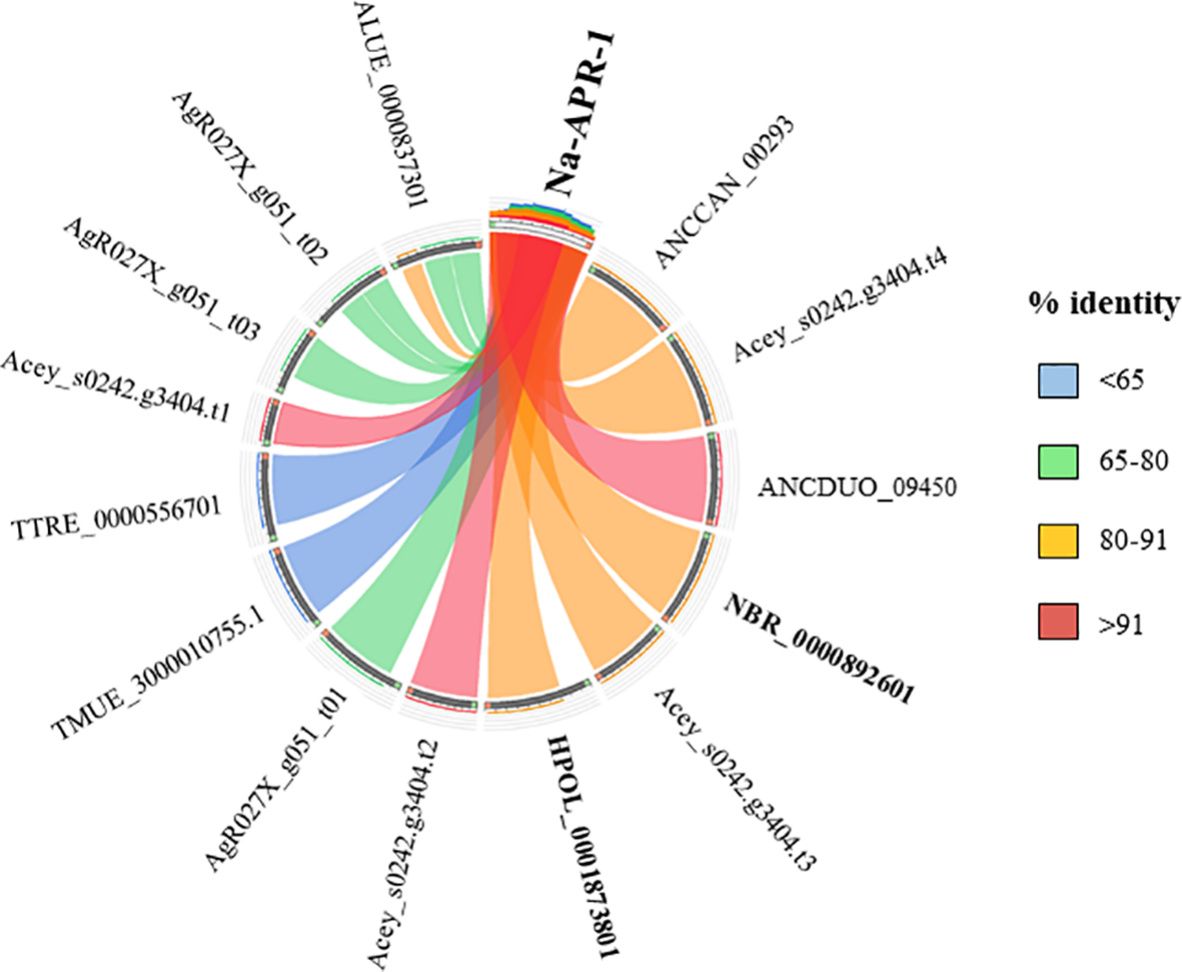
Similarity plot. Circos plot generated using Circoletto (Darzentas, 2010) showing the percentage of identity between Na-APR-1 and their homologues in different rodent model nematodes. Only homologues with e-values < 1E-50 are shown. Protein names as per Parasite WormBase database (v.15) have been used for comparison. ALUE, *Ascaris lumbricoides*; AgR, *Ascaris suum*; ANCCAN, *Ancylostoma caninum*; Acey, *Ancylostoma ceylanicum*; ANCDUO, *Ancylostoma duodenale*; HPOL, *Heligmosomoides polygyrus*; NBR, *Nippostrongylus brasiliensis*; TMUE, *Trichuris muris*; TTRE, *Trichuris trichiura*.

Currently there are no licensed vaccines against human STH, and The Human Hookworm Vaccine initiative is, at present, the only vaccine for hookworm infection in clinical development. This vaccine contains two recombinant antigens, Na-GST-1 and Na-APR-1, both key enzymes involved in the capacity of hookworms to use host blood as source of nutrients (Hotez et al., 2013). Furthermore, challenge studies conducted in laboratory animals have shown the capacity of Na-GST-1 and Na-APR-1 to induce protective efficacy (Hotez et al., 2010; Hotez et al., 2013). Interestingly, both proteins have homologues in other hookworm and hookworm-like parasites, with the highest homology found with *A. ceylanicum*, *A. caninum* and *A. duodenale* (**Figures 3** and **4**) as expected due to closeness of species. Homology found with mice models such as *H. polygyrus bakeri* and *N. brasiliensis* was also high, particularly for Na-APR-1, with >83% aminoacid identity in homologues from both parasites (**Figure 4**). Interestingly, the percentage of identity found with *Trichuris* spp. was ~60% while it was ~80% for *Ascaris* spp., suggesting a key role of this enzyme in ascarids, most likely due to these parasites potentially being blood-feeders (Toh et al., 2010). This could be of interest when ranking and selecting potential candidates against Ascaris infection, and a modified Na-APR-1 could be incorporated into a pan-anthelminthic vaccine as discussed by other authors (Zhan et al., 2014). In the case of Na-GST-1, >65% aminoacid identity was found in different homologues from both mice models (**Figure 3**), while similarity with *Ascaris* spp. was ~50%, which hampers its use as a vaccine candidate in other nematodes as discussed below for Trichuris.

The high similarity found between both vaccine candidates and their *N. brasiliensis* homologues has highlighted the conservation in the blood-feeding pathways with *N. americanus* (Bouchery et al., 2018). Indeed, vaccination with both hookworm vaccine candidates induced protection against *N. brasiliensis* in mice, which made authors suggest that *N. brasiliensis* is a suitable model for vaccine identification and drug screening against hookworms (Bouchery et al., 2018). On the other hand, the fact that *H. polygyrus bakeri* has homologues to these two proteins is intriguingly, since this parasite is believed to feed on epithelial cells and not on blood (Bansemir and Sukhdeo, 1994), and more experiments should be done to ascertain the role of Na-APR-1 and Na-GST-1 homologues in this hookworm-like model. Indeed, vaccination with GST in a mouse model did not confer protection against *H. polygyrus bakeri*, despite eliciting a significant humoral response (Brophy et al., 1994). Thus, it is tempting to speculate that GST might be a potential vaccine candidate only in blood-feeding nematodes, whereas in non-hematophagous nematodes, where this protein is suggested to play a role only as a defence mechanism against toxic substances (Smith, 1992), other candidates must be tested. As mentioned earlier, >60% sequence identity between two proteins usually results in similar functions; however, performing functionality studies and integrating different *omic* technologies is essential to obtain a more holistic picture of the biological problem.

It is also worth highlighting that different studies using experimental infection with helminths show that GST influences the immune responses and cross-reactive allergy (Mitchell, 1989; Smith, 1992; Brophy et al., 1995; Santiago et al., 2012). Furthermore, helminth and cockroach GST cross-react because of their noteworthy molecular and structural similarities, which has led several authors to suggest that vaccine development should take into account the potential impact of cross-reactivity with common allergens (Santiago et al., 2012). In this regard, it is also necessary to consider the ability of vaccines to induce strong Th2 responses, remembering the case described by Diemert et al. (Diemert et al., 2012) where generalized urticarial reactions were developed in several volunteers after vaccination with a single dose of Na-ASP-2. These allergic reactions were linked to pre-existing Na-ASP-2-specific IgE probably induced by previous infection with *N. americanus* (Diemert et al., 2012; Diemert et al., 2018).

Different studies have used the *N. brasiliensis* rodent model to (i) discover new vaccine candidates that could be extrapolated to human hookworm infections and (ii) develop novel administration routes of known vaccine candidates to improve their immunogenicity and reduce undesirable effects. Indeed, since *N. brasiliensis* has a highly conserved orthologue of Na-APR-1 (Bartlett et al., 2020), Bartlet et al. designed a lipopeptide-based vaccine using a B cell epitope derived from Na-APR-1, attached to a T helper epitope and administered it orally. In this study, several lipidated peptides were obtained and tested for vaccine efficacy using the *N. brasiliensis* hookworm model (Bartlett et al., 2020).

In previous studies, other researchers assessed the use of *N. brasiliensis* as a suitable model for testing vaccine candidates for hookworm infections. Using recombinant acetylcholinesterase B (AChE “B”), the most abundant enzyme isoform secreted by *N. brasiliensis* adult worms (Edwards et al., 1971; Clare Blackburn and Selkirk, 1992), these authors reported a level of protection in AChE-vaccinated animals and concluded that AChE “B” could be considered as a suitable vaccine antigen, with intranasal delivery being the most effective (Ball et al., 2007). Furthermore, the activity of the recombinant enzyme and subtypes of AChE in the somatic extract of *N. brasiliensis* could be inhibited by serum antibody (Ball et al., 2007); however, despite the promising results, no further studies pursued the development of a vaccine using this recombinant protein.

Cystatins, a group of proteins with immunomodulatory properties secreted by helminths, are implicated in several biological and pathological processes such as antigen processing, protein catabolism, and inflammation (Hartmann et al., 1997). Furthermore, cystatins have been identified in numerous parasite species including *N. brasiliensis*, where mice immunized with recombinant nippocystatin became partially resistant to infection, suggesting that *N. brasiliensis* might evade the host defense system using this protease inhibitor (Dainichi et al., 2001), although no other studies have tried to develop this molecule into a vaccine candidate in *N. brasiliensis* or other hookworms.

Coakley et al. showed that extracellular vesicles (EVs) from *H. polygyrus bakeri* are internalized by macrophages and can suppress host macrophage activation and inhibit expression of the IL-33 receptor subunit ST2. Further, vaccination with EVs elicited a protective immunity against *H. polygyrus bakeri* challenge in mice, suggesting EVs might play an important role *in vivo* (Coakley et al., 2017). The similarity of the EV proteomes between *H. polygyrus bakeri* and *N. americanus* is yet to be determined since EVs from the human hookworm have not been characterized yet.

*T. muris* is a well-established model for host immunity. Chronic infections using this model are obtained by a high-dose infection in the susceptible mouse strain AKR or by a low-dose infection in C57BL/6 mice. Furthermore, this model is widely used for assessing the efficacy and immunogenicity of vaccine antigen candidates against whipworm infections (Boes and Helwigh, 2000; Hurst and Else, 2013). Indeed, vaccination with *T. muris* ES products has been shown to elicit protective immunity in murine models (Jenkins and Wakelin, 1977; Jenkins and Wakelin, 1983; Dixon et al., 2008; Dixon et al., 2010; Liu et al., 2017). Furthermore, a recent study showed that immunisation with *T. muris* ES proteins stimulates long-lasting protection against a subsequent low dose infection, which naturally results in chronic infections (Shears et al., 2018). In this study, 11 potential immunogenic proteins were identified, including serpin, TCTP, GSCP and iPMG, all of which have direct homologues in *T. trichiura* (Shears et al., 2018) and could potentially be developed against the human whipworm. Despite these results constitute a great advance in the quest for a vaccine against *T. trichiura*, translation of these molecules into an effective treatment against the human whipworm will be challenging and further studies are needed.

Furthermore, several authors reported the identification of a whey acidic protein in the ES products from *T. muris*, *Tm*-WAP (r*Tm*-WAP49) (Briggs et al., 2018). In this study, the *Tm*-WAP protein was used to evaluate immunogenicity and protective efficacy in a *T. muris* infection mice model and determined that recombinant WAP protein (r*Tm*-WAP49) induces strong type 2 protective immunity (48% worm burden reduction). These authors also confirmed *Tm*-WAP is a potent immunodominant antigen abundantly secreted by *T. muris* adult worms and that recombinant *Tm*-WAP does not elicit antigen-specific IgE response. Furthermore, in this study the immunogenicity of the protein expressed with a Na-GST-1-tag (r*Tm*-WAP-F8+Na-GST-1) was shown to be protective (38% protection) in the susceptible AKR strain, although protection was related to the WAP fragment and not to the GST tag (Briggs et al., 2018), which could reflect the unessential requirement of blood in the parasite’s feeding process and impact a potential pan-nematode vaccine using this enzyme as discussed above.

In the case of Ascaris, *A. suum* has been widely used to assess the protection efficacy of different recombinant proteins in a mouse model of infection, and at least 5 candidates have been characterized to date (i.e. As14,As16, As24, As37 and As-Enol-1), all of them having direct homologs in *A. lumbricoides* (reviewed in (Zhan et al., 2014). While As-Enol-1 was developed as a DNA vaccine, having 61% efficacy in terms of larval recovery (Chen et al., 2012), As14,As16, As24 and As37 were tested in recombinant form and elicited a significant protection against subsequent infection ranging from 58-69% (Tsuji et al., 2001; Tsuji et al., 2002; Tsuji et al., 2003; Islam et al., 2005). Despite As-GST-1 has been proposed as a potential candidate mainly due to its homology (>50%) to Na-GST-1 at the aminoacid level (Liebau et al., 1997), its high allergenicity will have an important impact for the design on an anti-Ascaris vaccine (Acevedo et al., 2013). Importantly, since *A. lumbricoides* feeds on the host’s luminal content and not on blood, we might also speculate that this protein will not be part of the blood-feeding detoxification pathway, which might hamper its use as a vaccine, similarly to what occurs in *H. polygyrus bakeri* as described above.

## Using Murine Models for the Discovery of Novel Immunomodulators

Despite the significant harm caused by parasitic worms, numerous investigations have shown the faculty of helminths, and hookworms in particular, to modulate inflammation and their potential to treat inflammatory diseases (Croese, 2006; Feary et al., 2009; Croese et al., 2015). Indeed, different authors have suggested that allergies and autoimmune disorders are a consequence of our altered and reduced exposure to infectious antigens, including helminths (Wills-Karp et al., 2001; Yazdanbakhsh, 2002; Rook, 2005; Maizels and Nussey, 2013). Chronic hookworm infections are characterized by a robust and enduring Th2 cell response, and infected individuals do not show any signs of allergy and are, in fact, protected from developing allergies (Scrivener et al., 2001). In the case of hookworm models, rodents develop similar Th2 responses as observed in humans, and different studies have demonstrated the role of secreted proteins and other molecules in the immunomodulatory processes (Hewitson et al., 2009). Since the secretome of *N. americanus* was unknown until very recently (Logan et al., 2020), the scientific community has put the focus on the proteins secreted by the rodent hookworms and other STHs for their potential therapeutic action against allergies and possibly other inflammatory and autoimmune diseases (**Table 1**) (van Riet et al., 2007), including inflammatory bowel disease (IBD), type 1 diabetes, celiac disease and others (Helmby, 2015; Smallwood et al., 2017).

**Table 1:** Immunomodulatory molecules expressed in the hookworm models *Heligmosomoides polygyrus bakeri* and *Nippostrongylus brasiliensis*.

Molecule and description	Specie	Function	Reference
Hp-TGM	*H. polygyrus bakeri*	Ligation of TGF-β receptor on T cells leading to induction of Treg cells	(Grainger et al., 2010; Johnston et al., 2017)
HpARI	*H. polygyrus bakeri*	Blocks human and mouse IL-33	(Osbourn et al., 2017; Chauché et al., 2020)
HpBARI	*H. polygyrus bakeri*	Blocks the receptor of IL-33	(Vacca et al., 2020)
HpBARI_Hom2	*H. polygyrus bakeri*	Blocks the receptor of IL-33	(Vacca et al., 2020)
Calreticulin	*H. polygyrus bakeri*	Promotes Th2 cell-responses by interacting with scavenger receptor A	(Rzepecka et al., 2009)
EVs	*H. polygyrus bakeri*	Suppresses host macrophage activation and inhibits expression of the IL-33 receptor subunit ST2.	(Buck et al., 2014; Coakley et al., 2017)
EVs	*N. brasiliensis*	Suppresses inflammatory cytokines and increases expression of IL-10	(Eichenberger et al., 2018a)
Cystatin (HpCPI)	*H. polygyrus bakeri*	Modulates differentiation and activation of BMDCs resulting in non-functional dendritic cells.	(Sun et al., 2013)

Hp-TGM, TGF-β mimic; HpARI, Alarmin release inhibitor; HpBARI, H. polygyrus Binds Alarmin Receptor and Inhibits; EVs, extracellular vesicles.

Human trials using live hookworm infections have been and are currently in development, but present strong limitations and challenges such as cost, reproducibility and ethical issues. Consequently, animal models are invaluable research tools that might provide new knowledge about the individual molecules involved in the immunomodulatory processes. For instance, the 41 kDa neutrophil inhibitory factor (NIF) and the tissue inhibitors of metalloprotease Ac-TMP-2 (renamed as Ac-AIP-2) were characterised from *A. caninum* and have been shown to have important anti-inflammatory properties (Xu et al., 2000; Navarro et al., 2016). Similarly, a serine protease inhibitor from *T. suis* (TsCEI), as well as the proteins triosephosphate isomerase and nucleoside diphosphate kinase have been shown to have important immunomodulatory properties (Rhoads et al., 2000; Leroux et al., 2018). Other hookworm proteins and their immunomodulatory roles have been reviewed elsewhere (Abuzeid et al., 2020; Ryan et al., 2020).

The immunomodulatory role of *H. polygyrus bakeri* is indisputable. This role has been attributed, among others, to different secreted proteins, including three proteins that belong to the complement control protein (CCP) superfamily: Hp-TGM (*H. polygyrus bakeri* TGF-β mimic), HpARI (*H. polygyrus bakeri* Alarmin Release Inhibitor) and HpBARI (*H. polygyrus bakeri* Binds Alarmin Receptor and Inhibits). Hp-TGM has been shown to drive Treg production in mice and humans by binding to the mammalian TGF-β complex, despite it has no sequence homology to mammalian TGF-β (Johnston et al., 2017). Furthermore, treatment with rHp-TGM increased the number of Treg cells in draining lymph nodes at the site of graft transplant in mice, resulting in delayed allograft rejection (Grainger et al., 2010; Johnston et al., 2017). HpARI is a cytokine-binding protein that prevents alarmin release within necrotic cells by binding directly to IL-33 and nuclear DNA (Osbourn et al., 2017; Chauché et al., 2020). Indeed, intranasally-administered rHpARI suppressed eosinophil responses and ILC2s in the lungs of mice following the exposure to *Alternaria* allergen, while it increases worm burden and suppresses type 2 responses in *N. brasiliensis*-infected mice (Osbourn et al., 2017). It has also been shown that *H. polygyrus bakeri* can block the IL-33 pathway by blocking the cytokine and its receptor *via* both HpARI and HpBARI, respectively (Vacca et al., 2020). The same authors also identified a close homologue of HpBARI (HpBARI_Hom2), which binds and inhibits the human form of the IL-33 receptor (Vacca et al., 2020). These discoveries highlight a potential use for the referred proteins in a wide variety of inflammatory settings, particularly in asthma (Chauché et al., 2020).

It is also remarkable that calreticulin from *H. polygyrus bakeri* has been shown to promote Th2 cell-responses but no further studies have explored into the immunomodulatory effects of this molecule (Rzepecka et al., 2009).

Although cystatins have been used as vaccination targets, rHp-CPI from *H. polygyrus bakeri* is also capable to modulate the activation and differentiation of bone-marrow-derived CD11c+ DC (BMDC), and to interfere with antigen and MHC-II molecule processing and Toll-like receptor signalling pathway, resulting in functionally deficient dendritic cells that induce a suboptimal immune response in mouse models. (Sun et al., 2013).

In addition to individual molecules, the recent characterization of EVs secreted by different nematodes has highlighted their potential role as immunomodulators. Administration of *H. polygyrus bakeri* EVs reduced lung immunopathology by modulating innate immunity *via* suppression of the early IL-33 and the later type 2 (specially ILC2) allergic responses (Buck et al., 2014). Furthermore, *N. brasiliensis* secreted EVs suppressed the production and secretion of proinflammatory cytokines and increased the expression of IL-10, protecting mice from T-cell-dependent induced colitis (Eichenberger et al., 2018a).

In conclusion, the latest advances have highlighted the similarities between several human nematodes and their respective murine models at a genomic and proteomic level. These results highlight the suitability of these models, not only for the study of the immune responses associated to infection with STHs, but also, in some cases, for the development of new vaccine candidates and immunomodulatory molecules. However, further research should aim at integrating the different available *omic* technologies (e.g. transcriptomic, proteomic, metabolomics and lipidomic, among others) to obtain a more comprehensive picture of the biology of these worms and confidently validate candidate molecules.

## Author Contributions

KM, CC, and JS analyzed the data, wrote, and edited the manuscript. All authors contributed to the article and approved the submitted version.

## Funding

JS is a Miguel Servet Fellow funded by Instituto de Salud Carlos III (CP17III/00002, MPY 406/18 and MPY 504/19). The funders had no role in study design, analysis, decision to publish, or preparation of the manuscript.

## Conflict of Interest

The authors declare that the research was conducted in the absence of any commercial or financial relationships that could be construed as a potential conflict of interest.
